# Counter-regulatory RAS peptides: new therapy targets for inflammation and fibrotic diseases?

**DOI:** 10.3389/fphar.2024.1377113

**Published:** 2024-04-10

**Authors:** Diana V. Ávila-Martínez, Wendy K. Mixtega-Ruiz, José M. Hurtado-Capetillo, Oscar Lopez-Franco, Mónica Flores-Muñoz

**Affiliations:** ^1^ Laboratorio de Medicina Traslacional, Instituto de Ciencias de la Salud, Universidad Veracruzana, Xalapa, Mexico; ^2^ Doctorado en Ciencias de la Salud, Instituto de Ciencias de la Salud, Universidad Veracruzana, Xalapa, Mexico; ^3^ Doctorado en Ciencias Biológicas, Centro Tlaxcala de Biología de la Conducta, Universidad Autónoma de Tlaxcala, Tlaxcala, Mexico; ^4^ Centro de Estudios y Servicios en Salud, Veracruz, Mexico

**Keywords:** inflammation, fibrosis, counter-regulatory RAS, angiotensin type 2 receptor, Mas receptor

## Abstract

The renin-angiotensin system (RAS) is an important cascade of enzymes and peptides that regulates blood pressure, volume, and electrolytes. Within this complex system of reactions, its counter-regulatory axis has attracted attention, which has been associated with the pathophysiology of inflammatory and fibrotic diseases. This review article analyzes the impact of different components of the counter-regulatory axis of the RAS on different pathologies. Of these peptides, Angiotensin-(1–7), angiotensin-(1–9) and alamandine have been evaluated in a wide variety of *in vitro* and *in vivo* studies, where not only they counteract the actions of the classical axis, but also exhibit independent anti-inflammatory and fibrotic actions when binding to specific receptors, mainly in heart, kidney, and lung. Other functional peptides are also addressed, which despite no reports associated with inflammation and fibrosis to date were found, they could represent a potential target of study. Furthermore, the association of agonists of the counter-regulatory axis is analyzed, highlighting their contribution to the modulation of the inflammatory response counteracting the development of fibrotic events. This article shows an overview of the importance of the RAS in the resolution of inflammatory and fibrotic diseases, offering an understanding of the individual components as potential treatments.

## 1 Introduction

Inflammation is a physiological process used as a defense mechanism in response to tissue injury and infection. When an infection or tissue injury triggers an acute inflammatory response, blood components (proinflammatory mediators) are released to the local site where the signal begins. These signals are sent by endothelial cells, tissue-resident macrophages, and, in some tissues, mast cells, resulting in the recruitment of immune cells that orchestrate actions eliminating harmful stimulus and promoting tissue repair. If stimuli are not resolved and repaired, protein excess sets in the extracellular matrix causing the destruction of the original tissue architecture, originating fibrosis that leads to organ malfunction. The main effector cells in fibrosis, myofibroblasts, are responsible for remodeling the extracellular matrix. Increasing evidence has demonstrated that a major pathway in fibrosis generation is the transforming growth factor-β (TGF-β) and, indeed, this factor promotes a sustained fibrogenic immune cell phenotype (Frangogiannis, 2020).

Over the last years, researchers have discovered that the renin-angiotensin system (RAS), besides having an important role in the regulation of electrolyte balance, intravascular volume, and blood pressure, is also implicated in triggering inflammation and promoting tissue remodeling. Emerging evidence supports that the peptides from the newly discovered counter-regulatory arm of RAS, as part of the opposite effects to classic RAS, may also elicit anti-inflammatory and antifibrotic effects.

**FIGURE 1:**
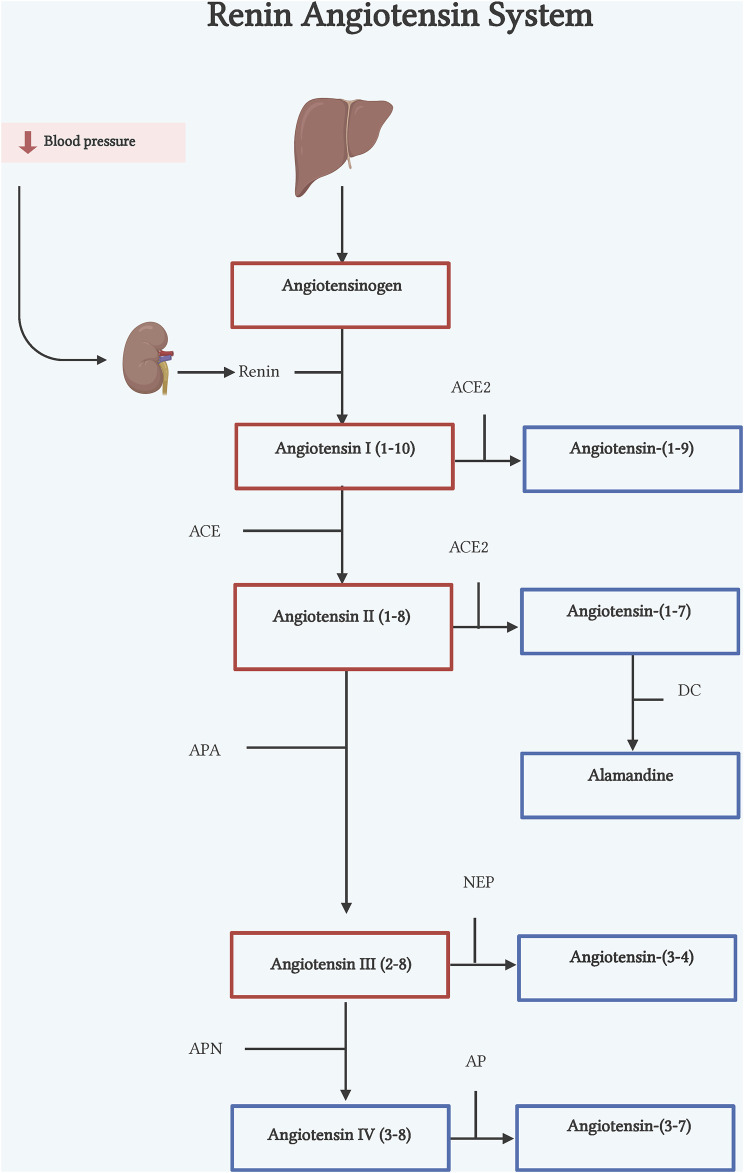
Renin-angiotensin system. Classic axis. Angiotensinogen (AGT), synthesized in the liver, is cleaved by renin, an enzyme synthesized by the kidneys, to form Ang I. Ang I is cleaved by ACE to form Ang II. Counter-regulatory axis. Ang I and Ang II are cleaved by ACE2 to form Ang-(1–9) and Ang-(1–7) respectively. Ang II is also cleaved by APA to form Ang III, by NEP to form Ang-(3–4), and by APN to form Ang IV. DC, decarboxylase; APA, aminopeptidase A; NEP, neutral endopeptidase; APN, aminopeptidase N; AP, aminopeptidase. Illustration was created with BioRender.com.

In this review, we describe some of the mechanisms were the counter-regulatory peptides have been found to be regulatory elements in inflammation and fibrotic processes, making them an interesting therapeutic target (Figure 1).

## 2 From inflammation to fibrosis

The initial recognition of pathogens or cell damage by tissue macrophages through pattern recognition receptors (PRRs), release a variety of cytokines, chemokines, and eicosanoids [such as tumor necrosis factor-alpha (TNF-α), interleukine (IL) 6, IL-1β, monocyte chemoattractant protein 1 (MCP-1), IL8, prostaglandins, *etc.*,] allowing the recruitment and transmigration of neutrophils, monocytes, and dendritic cells. Transcription nuclear factor kappa B (NF-κB) is one of the most common pathways activated by PRRs, which is responsible for inflammatory mediators’ induction by different immune cell types (Mussbacher et al., 2019). In activated M1 macrophages, NF-κB is a key pathway required for a large number of inflammatory genes including IL-1β, IL-6, IL-12, TNF-α, and cyclooxygenase-2 (Chen S. et al., 2023). Furthermore, NF-κB activation plays an important role in T cell activation and differentiation, thus, the pathway induces Th1 polarization improving the induction of cytokines and antigen presentation (Liu et al., 2017).

The transmigration mechanism is mediated by selectins (P, L, and E selectins) and integrins which facilitate adhesive interactions between leukocytes and endothelial cells of blood vessel walls, and additional chemokines and lipid chemoattractants to govern the migration of leukocyte route to inflamed tissue (Nourshargh and Alon, 2014). Inside the infection site, pathogens are opsonized, and the activated neutrophils attempt to kill the pathogens by releasing reactive oxygen species (ROS), reactive nitrogen species, proteinase 3, and cathepsin, found in the content of their granules. Consequently, surrounding tissue impairs the release of mediators that, not only contribute as inflammation signals but, also, triggers an antifibrinolytic coagulation cascade. The circulating platelets bind to exposed collagen and von Willebrand factor, activating and leading to the production of growth factors. Beyond this, other coagulation factors help to form fibrin and blood clots. (Serhan and Savill, 2005).

If the insult is eliminated, the set of signals switches from pro-inflammatory to anti-inflammatory cues such as lipoxins, resolvins, prostaglandins, and anti-inflammatory cytokines such as TGF-β or IL-10 (Levy et al., 2001; Serhan and Savill, 2005). The excess cells that had to proliferate in response to inflammation and damaged endothelial cells, now undergo apoptosis as an essential step for the clearance of resolved inflammation. During this procedure, suppression of NF-κB in neutrophils has been seen, which increases the cytotoxic effects of TNF-α (Ward et al., 1999). Apoptotic cells are phagocytized by a process called efferocytosis which includes the beginning of tissue restoration (Schmid and Brüne, 2021). The repair process involves the replacement of injury cells and components of extracellular matrix (ECM) that are beneficial for the healing process. As already mentioned, when the platelets are activated, they produce growth factors and through TGF-β action, stimulate fibroblast infiltration and its subsequent differentiation into myofibroblast, which promotes the production of collagen deposits and wound healing. In physiological conditions, ECM components are turnover and degraded by a family of proteinases termed matrix metalloproteinases (MMP) (Nathan and Ding, 2010).

Persistent tissue damage and non-resolving inflammation contribute to the development of chronic inflammation, leading to excessive deposits of ECM components generating fibrosis. Hence, fibrosis has been defined as the accumulation of ECM, mainly collagen and fibronectin, around damaged tissue. But the main question is, why is there persistent tissue damage? There are numerous reasons for that, for instance persistent infections by *Helicobacter pylori* (Waluga et al., 2015; Zahmatkesh et al., 2022), Schistosomes (Zhong et al., 2022), hepatitis viruses (Zhou et al., 2021), SARS-COV-2 virus (Shen et al., 2022), recurrent exposure to toxins, allergic and asthma (Hough et al., 2020), obesity, diabetes (Xiong et al., 2022) and hypertension (Schimmel et al., 2022).

## 3 The renin-angiotensin system and its counter-regulatory arm

The classical pathway of RAS begins with cleavage of the 10 N-terminal amino acids of angiotensinogen (ANG) by the enzyme renin, converting it into angiotensin I (Ang I) (Lu et al., 2016) (Figure 1). Renin is the active enzyme produced by juxtaglomerular cells in the kidney and released into circulation in response to sympathetic activation, low levels of sodium in renal tubules, and low pressure in afferent arterioles of the renal glomerulus (Muñoz-Durango et al., 2016). Human ANG is a glycoprotein that is composed of 485 residue amino acids. The liver is considered the main source of ANG, but other organs and cells such as the heart, kidney, brain, and leukocytes can express ANG in pathophysiological conditions (Gomez et al., 1993; Tamura et al., 1998; Kobori et al., 2007; Klimov et al., 2012). Subsequently, the angiotensin-converting enzyme (ACE), expressed in many tissues, cleaves the C-terminal dipeptide from Ang I to produce Angiotensin II (Ang II) octapeptide. Though, *in vitro* ACE activity also hydrolyzes other peptides such as bradykinin, N-acetyl-Ser-Asp-Lys-Pro and Angiotensin-(1–7) [Ang-(1–7)] due to its two catalytic sites (Hooper and Turner, 2003) (Figure 1).

Ang II is the most studied components of RAS, known for its principal effects as a vasoactive mediator. Ang II binds to its two main receptors, the Ang II type 1 and 2 receptor (AT_1_Rand AT_2_R, respectively). Both receptors are part of the G protein-coupled receptor family. AT_1_R mediates the main roles of Ang II in blood pressure regulation and body fluid homeostasis. Whereas AT_2_R activation is considered to have antagonist effects to AT_1_R. However, its complete role in different pathologies is still being studied. There are several reports describing an increase in the levels of several components of the RAS in different diseases such as hypertension, progressive nephropathic disease, diabetes, septic shock, asthma, aneurism, aging, and acute respiratory distress syndrome (Millar, et al., 1995; Kuba et al., 2006; Chawla et al., 2010; Antonucci et al., 2017), suggesting a wider effect of RAS, than merely control of blood pressure.

**FIGURE 2:**
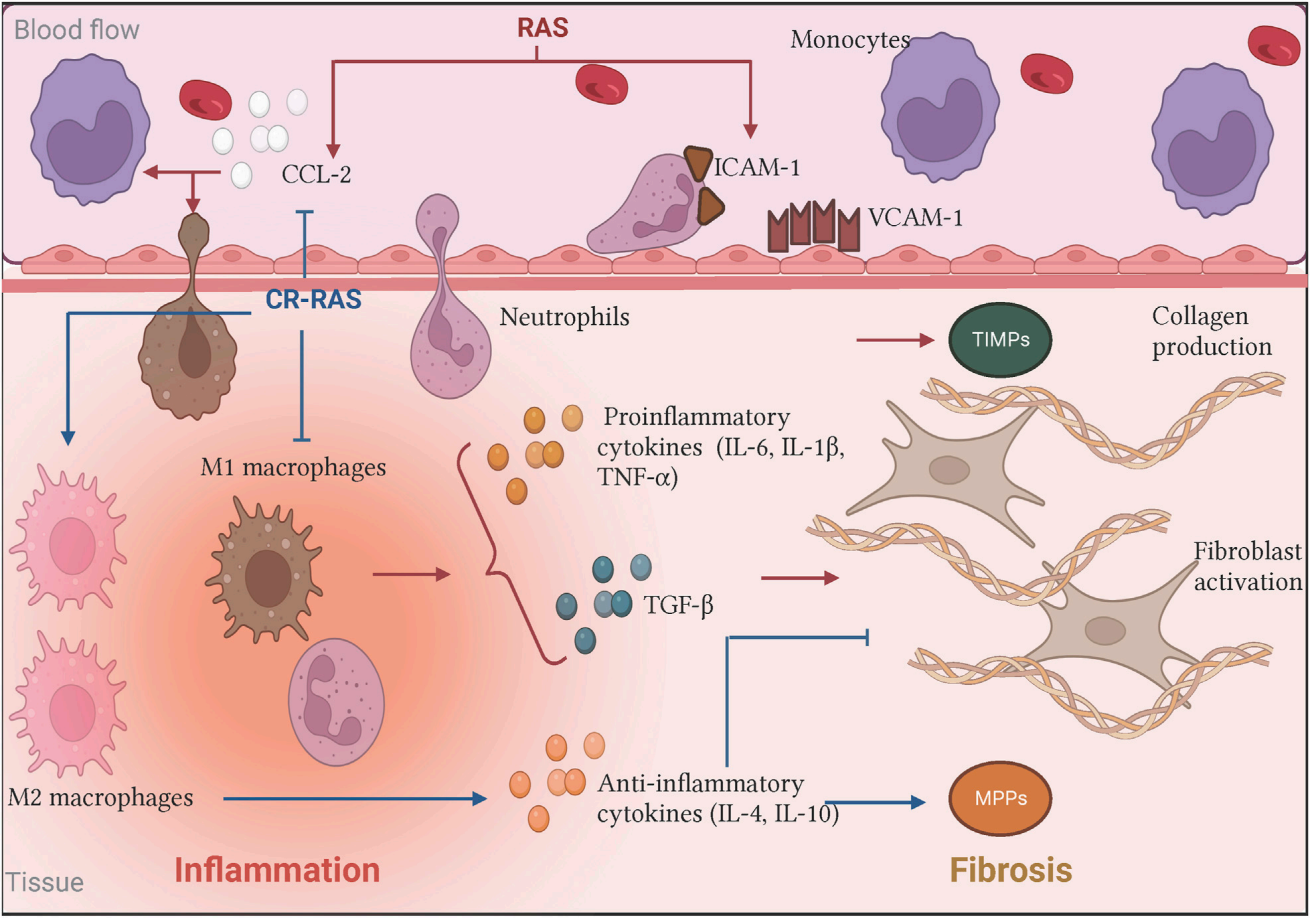
Biological effects of the RAS and the Counter-regulatory RAS on inflammation and fibrosis. In the classical axis, Ang II stimulus (illustrated as RAS in red bold letters) increases CCL-2 production and upregulates adhesins expression. Moreover, Ang II promotes pro-inflammatory cytokines releasing such as IL-6, which induces immunological cell recruitment and promotes its activation favoring the inflammatory process. In turn, the persistent signal gives rise to immune cells producing other mediators creating a microenvironment where the inflammation did not resolve, contributing to fibrogenesis by fibroblast activation and subsequent excessive collagen deposition. On the other hand, some of the counter-regulatory RAS (illustrated as CR-RAS in blue bold letters) have demonstrated opposite effects. Illustration was created with BioRender.com.

In the last decades, Ang II´s proinflammatory effects linked to fibrosis have been studied. Ang II is capable of up regulating many inflammatory mediators. Ang II infusion in normotensive subjects increased IL-6 plasma levels (Luther et al., 2006). Likewise, Ang II-infused mice showed an increase in the expression of cell adhesion proteins VCAM-1 and ICAM-1 on the surface of endothelial and leukocyte cells respectively, playing an important role in leukocyte adhesion (Qiu et al., 2022). Furthermore, Ang II raised IL-1β, IL-6, TNF-α levels, and macrophage infiltration in heart and vessel walls. These changes boosted TGF-β expression and collagen I/III production, promoting subsequent cardiac remodeling (Zhang et al., 2023). These effects were also observed in hypertensive patients with heart failure (Muñoz-Durango et al., 2016), suggesting that blocking inflammatory proteins could attenuate hypertensive cardiac remodeling (Wenzel et al., 2011; Qiu et al., 2022; Zhang et al., 2023). Moreover, expression of endoglin, a type III TGF-β receptor, and endothelin-1 in cardiac fibroblast (CF) were increased after Ang II treatment (Chen et al., 2004). Ang II also elicits an increase in collagen protein expression and a decrease in MMP-1 protein. These effects were abolished by AT_1_R and MAPp42/44 inhibitors, whereas AT_2_R antagonists had no effect (Chen et al., 2004). In addition, Ang II-induced TGF-β and endothelin-1 expression in CF promoted cardiac hypertrophy and renal fibrosis *in vitro* and *in vivo* (Gray et al., 1998; Sun et al., 2000; Schultz et al., 2002; Chen et al., 2004). This evidence implies that Ang II could promote profibrotic responses up-regulating TGF-β and endothelin-1 expression through the AT_1_R/MAPp42/44 pathway (Figure 2).

In addition, there is evidence of RAS overactivation in adipose tissue in obesity. It has been shown that Ang II induces insulin resistance and favors inflammation (Goossens et al., 2007). Ang II *in vitro* treatment increases MCP-1 secretion and proinflammatory cytokines. In contrast, adipocytes produce low levels of IL-10 after peptide stimuli, which impairs insulin secretion (Kalupahana et al., 2012). Moreover, pancreatic islet exposure to Ang II also induces cytokine production, impairs insulin secretion, and triggers β cells apoptosis. Interestingly, cytokine IL-1β blocking reduced inflammation, and restored insulin secretion, suggesting that inflammation deteriorates pancreatic islet function and could lead to end-organ damage (Sauter et al., 2015). On the other hand, scientific evidence has investigated the role of RAS in aging (Takeshita et al., 2023). Ang II has been implicated in the senescence process. The Ang II *in vitro* stimuli of human umbilical endothelial vein cells (HUVEC) and vascular smooth muscle cells (VSMC) induced cell growth arrest, and increased senescence-associated β-galactosidase, observing fragmented nuclei, and increase in apoptotic cells (Shan et al., 2008). Furthermore, persistent Ang II stimulation upregulates senescence-related protein expression such as p21, p16, p27, and p53 including a boost in ROS production and activation of transcription factors NF-κB and AP-1 in VSMC. These effects were inhibited by AT1R and PI3K/Akt inhibitors, suggesting that Ang II could exhibit its effect by AT1R/PI3K/Akt/p53/p21 pathway (Min et al., 2007; Li et al., 2014).

In addition to Ang II, other identified angiotensin peptides have been described as having counter effects to the classical RAS pathway. This non-classical pathway is also called the counter-regulatory arm and comprises several peptides product of Ang II and its precursor´s cleavage. The counter-regulatory RAS was identified through the concept of a local RAS based on two discoveries: first, expression of all components of the RAS in specific tissues. ACE, renin, angiotensinogen, Ang I and Ang II are expressed and synthesized in human and murine cardiomyocytes (Paul et al., 1993; Hirsch et al., 1991; Burrell et al., 2005; van Kats et al., 1998). Both the AT_1_R and the AT_2_R have also been shown to be expressed in cardiac cells (Booz and Baker, 1996). All these findings suggested that generation and consequently Ang II-mediated signaling could be initiated directly in individual tissues without the requirement for systemic, circulating RAS components. The second discovery was the identification of new components of the RAS expressed in specific tissues, predominantly the enzyme Angiotensin-converting enzyme 2 (ACE2). These findings resulted in the theory of a “local and tissue-specific” RAS. Since the recognition of this local tissue-specific RAS many studies have been described to elucidate the actions and mechanisms of their components and to attempt to segregate these actions from those mediated by circulating Ang II produced via the classical pathway. This has led to the characterization of the physiological and pathophysiological action of the RAS in different organs. Local tissue-specific RAS has been described in the heart, blood vessels, kidney, adrenal gland, nervous system, reproductive system, skin, digestive system, lymphatic and adipose tissue and in fetal development.

One of the main discoveries of the counter-regulatory RAS is the generation of Angiotensin peptide metabolites. These peptides include Ang-(1–7), angiotensin-(1–9) [Ang-(1–9)], angiotensin-(3–7) [Ang-(3–7)], angiotensin-(3–4) [Ang-(3–4)], angiotensin-(1–5) [Ang-(1–5)], angiotensin-(2–8) [Ang-(2–8)], angiotensin-(3–8) [Ang-(3–8)], also called angiotensin III (Ang III) and IV (Ang IV) respectively, and alamandine (Paz Ocaranza et al., 2020).

### 3.1 Angiotensin-(1–7)

Ang-(1–7) is a heptapeptide discovered in 1988 in the brainstem by Santos et al. (1988). The first characterization of the peptide described it as an Ang I´s metabolite by ACE enzymatic action. However, after ACE inhibition, Ang-(1–7) was still detectable, suggesting that other enzymes could generate this peptide (Santos et al., 1988). Some years later, ACE2 was identified as an ACE homolog, that was able to hydrolyze Ang II to Ang-(1–7) (Tipnis et al., 2000) and Ang I to Ang-(1–9) (Donoghue et al., 2000). Unlike ACE, ACE2 is tissue-specific, firstly described as expressed only in heart, kidney, and testis (Donoghue et al., 2000), to then be shown to express in many other tissues, as enterocytes, reproductive cells, eyes, etc. (Hikmet et al., 2020). Subsequently, Ang I can also be converted into Ang-(1–7), via either cleavage by ACE 2, to form Ang-(1–9) which is then hydrolyzed by ACE transforming it into Ang-(1–7), or by a direct conversion by the action of prolyl endopeptidase, neutral endopeptidase or thimet oligopeptidase (Welches et al., 1993) (Figure 1). Although it has been proved that ACE2 has higher catalytic efficiency on Ang II to form Ang-(1–7), its formation at least in failing human heart, has been reported also by NEP suggesting that its pathway generation is dependent of physiological conditions (Vickers et al., 2002; Zisman et al., 2003; Rice et al., 2004). Ang-(1–7) acts through engaging the orphan Mas proto-oncogene (Santos et al., 2003). However, there is evidence that shows Ang-(1–7) can also bind to AT_2_R although with less specificity (Bosnyak et al., 2011).

**FIGURE 3:**
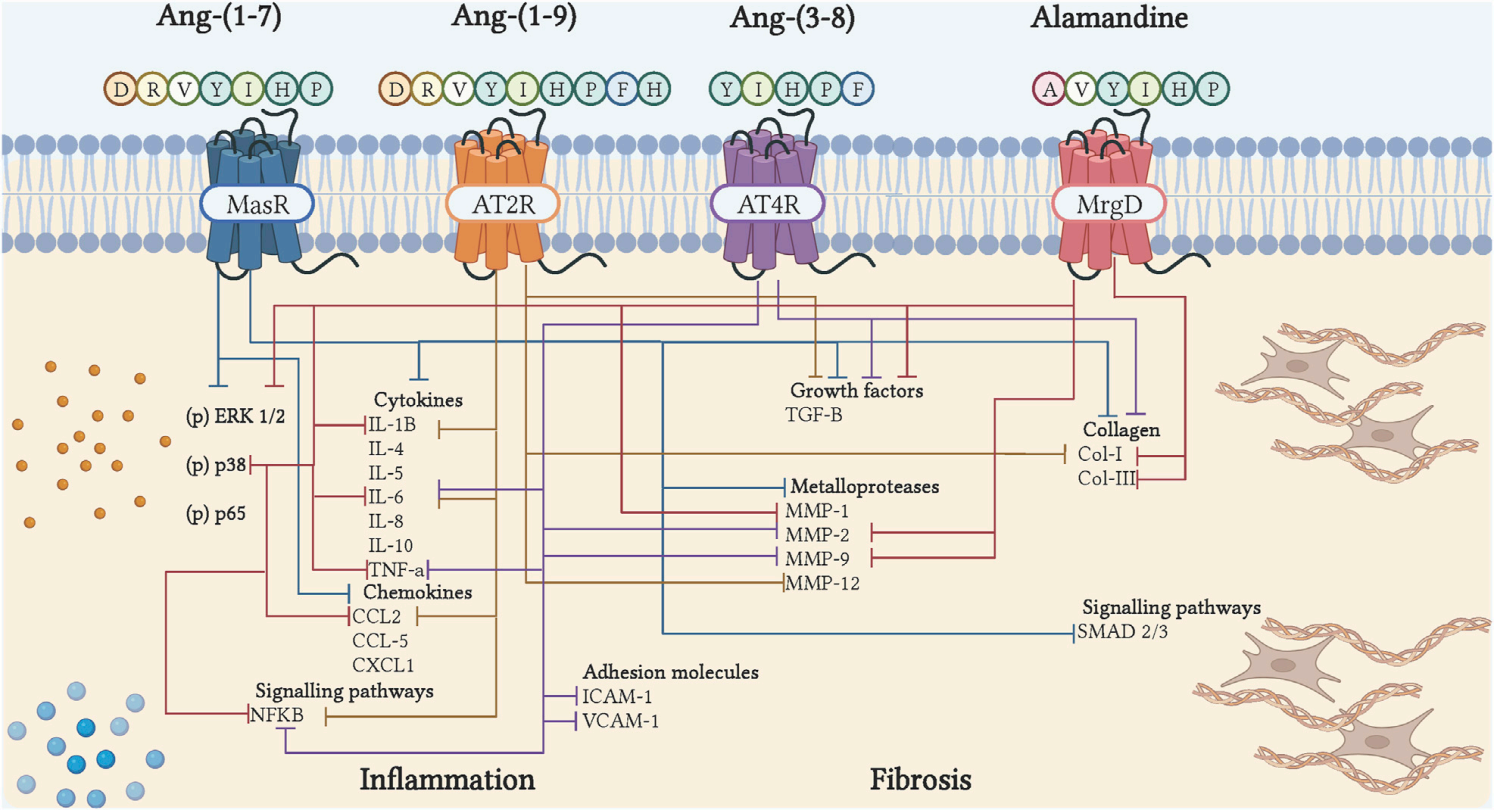
Cell signaling of bioactive peptides of the counter-regulatory axis in inflammation and fibrosis. The binding of peptides to their receptor induces inhibition of cytokines, chemokines, adhesion molecules, growth factors, collagen, metalloproteases, and inflammatory and fibrotic signaling pathways. The blue arrows show the actions of Ang-(1–7), through Mas; the orange arrows, showing the actions of Ang-(1–9), via AT_2_R; the purple arrows, show the actions of Ang-(3–8), through AT_4_R; the red arrows, show the actions of Alamandina, through MrgD. Illustration was created with BioRender.com.

The first described actions for Ang-(1–7) were in the cardiovascular system, where it inhibited Ang II-induced vasoconstriction (Roks et al., 1999; Jiang et al., 2014). Afterward, several studies have shown anti-inflammatory and antifibrotic effects in different pathological conditions (Figures 2, 3). There is evidence that Ang-(1–7) treatment alleviates induced inflammation by cecal ligation and puncture in murine models (Passaglia et al., 2023). Ang-(1–7) reduced expression of proinflammatory cytokines TNF-α and IL-6 while augmenting anti-inflammatory cytokines IL-4 and 10 and promoting M2 macrophage polarization. These effects were accompanied by a decrease in NF-kB phosphorylation, suggesting that Ang-(1–7) inhibits inflammation through this pathway (De Carvalho Santuchi et al., 2019; Pan et al., 2021). In another study using a *E. coli*-induced peritonitis, administration of Ang-(1–7) promoted the recruitment of M2 macrophages, increasing its phagocytic capacity, and increased the production of IL-10, MCP-1. These effects were inhibited in the absence of the Mas receptor, suggesting that Ang-(1–7) could contribute to the resolution of inflammation through the Mas receptor (Zaidan et al., 2022).

Furthermore, the role of Ang-(1–7) during sepsis has been evaluated. The infusion of this peptide prevented septic shock in different animal models, limiting the amount of IL-6 cytokine, nitric oxide (NO) production and decreasing renal, lung and liver damage, along with IκB kinase pathway inhibition, suggesting that Ang-(1–7) ameliorate sepsis-induced organ injury likely through the inhibition of an inflammatory response (Tsai et al., 2021; Garcia et al., 2023). In the septic process, an increase in Ang II levels has been shown and is associated with renal and myocardial injury with an increase in cytokine levels (Zhu et al., 2021; Chen X. S. et al., 2023). It has been found that administration of Ang-(1–7) decreases inflammatory cells infiltration as well as the production of IL-6, IL-1β, and TNF-α in myocardial and renal tissue of septic mice. Additionally, a higher survival percentage and a decrease of Ang II levels were observed in mice that were administered with Ang-(1–7) compared to untreated mice suggesting that treatment with this peptide could attenuate the organic damage caused by sepsis (Xu et al., 2021; Zhu et al., 2021; Chen X. S. et al., 2023).

During the recent coronavirus pandemic, researchers found that there was a RAS dysregulation and ACE2 dysfunction due to SARS-COV-2 binding to ACE2 receptor in host cells, driving to acute respiratory distress syndrome. COVID-19 patients exhibit reduced Ang-(1–7) levels compared to control individuals and this phenomenon was associated with the severe form of the disease (Carpenter et al., 2022). Therefore, the peptide was also suggested as a therapy in COVID-19 ill patients confirmed with high viral load and co-morbidities such as hypertension, diabetes, heart diseases and asthma. Plasma enriched with Ang-(1–7) was transfused in combination with favipiravir treatment and, in almost all cases, the peptide transfusion improved oxygen saturation and clinical status (Onal et al., 2022). It has been shown that SARS-COV-2 infect bronchoalveolar stem cells (BACS). BACS are a lung resident stem cell population that differentiate into many types of cells, contributing to the maintenance of bronchoalveolar duct. In response to injury, BACS ratio increases and promotes tissue repair, which is impaired when viral infection. In mice, Ang-(1–7) administration increased the percentage of BACS and decreased protein expression involved in programmed cell death and inflammation process, suggesting that Ang-(1–7) peptide treatment could have a protective function in lung injury by SARS-CoV-2 (Ciechanowicz et al., 2022). In addition, Ang-(1–7)´s *in vitro* treatment of human pulmonary alveolar epithelial cells stimulates with SARS-COV-2 spike protein reduced production of IL-6, IL-8 and suppressed ERK1/2 and AP-1 phosphorylation, suggesting that Ang (1–7) could block SARS-COV-2-exacerbating proinflammatory response (Shen et al., 2023).

On the other hand, there is experimental evidence indicating Ang-(1–7)/Mas pathway may contribute to the attenuation of airway pathological conditions. Asthma is a chronic allergic airway disease and is characterized by airway hyperresponsiveness and pulmonary remodeling in which inflammatory response play an important role with a predominance of eosinophils, increased IL-4, IL-5, IL-13, TNF-α cytokines, and chemokines such as MCP-1 and CCL5 (Magalhães et al., 2016). Ovalbumin challenge in mice is used as an experimental asthma model, since it replicates the pathophysiological features in patients (Magalhães et al., 2015). In previous studies using this model, Ang-(1–7) treatment statistically diminished the release levels of IL-4, IL-5 cytokines and MCP-1 and CCL-5 chemokines into serum, as well as granulocyte-macrophage colony-stimulating factor. Consequently, reducing inflammatory cell infiltrate, alveolar wall thickening and airway collagen I and mucus deposition (Magalhães et al., 2015). In addition, Ang-(1–7) administration upregulated Mas expression on bronchial cells and decrease epidermal growth factor receptor (EGFR), Src kinase and ERK1/2 phosphorylation in lung tissue (El-Hashim et al., 2019). Nonetheless, it has been demonstrated that Mas deficiency aggravates chronic allergic pulmonary inflammation, suggesting that Mas is an important receptor to trigger anti-inflammatory response and could be doing it through mediation of EGFR/Src/ERK1/2 pathway on lung tissue (Magalhães et al., 2016). Additionally, ATG5 is a key protein involved in the formation of autophagosomes overexpressed in lung tissue of asthma patients. Interestingly, Ang-(1–7) elicited decreased levels of ATG5 on human bronchial epithelial and smooth muscle cells. This ATG5 deficiency suppressed inflammation and airway fibrosis, similarly to what it was observed in ovalbumin-challenged mice with Ang-(1–7) administration, suggesting another mechanism for Ang-(1–7) as an asthma treatment (Xu et al., 2023).

As already mentioned, the end result of inflammation is fibrosis. In models of acute lung injury mimicking acute respiratory distress syndrome in humans, the action of Ang-(1–7) have been examined. In this syndrome, there is a disruption of epithelial and endothelial barriers in the lung leading to severe immune reactions, hypoxic respiratory failure, pulmonary edema, and fibrosis (Chen et al., 2013). In acute lung injury, the characteristic increase in the expression of collagen I and III, TGF-β, IL-6, and Smad2/3 in the lung, is reduced after Ang-(1–7) therapy, leading to a significant improvement in oxygenation and reduction of white blood cells counts mainly in neutrophil percentage (Chen et al., 2013; Zambelli et al., 2015; Wang et al., 2022). In addition, Ang-(1–7) infusion, starting immediately after lung injury, has also been shown to prevent pulmonary vascular resistance, diminish aortic pressure, and increase ACE2 activity in lung tissue (Supé et al., 2016). Ang II-induced pulmonary fibrosis in rats triggers a cascade of proinflammatory factors and adhesion molecules that allows infiltrate cells recruitment in the lung, mediated by phosphorylation of ERK1/2/NF-κB pathway. Interestingly, Ang-(1–7) treatment in Ang II-treated animals inhibited ERK1/2/NF-κB axis, proinflammatory cascade and ameliorated lung fibrosis, effects that were reverse by Mas antagonists. By contrast, in Ang-(1–7) alone group, exhibited lung inflammation and deposition of collagen I compared to the untreated group (Meng et al., 2014). Together these data showed, at least under pathological conditions, Ang-(1–7) can protect against fibrosis development down-regulating pathways such as ERK1/2/NF-κB, which makes it a therapeutic target in fibrotic conditions (Figure 3).

Ang-(1–7) has also been studied in fibrotic renal diseases. Renal diseases are characterized by interstitial cell infiltration, NF-κB activation, increased apoptosis, oxidative stress, and fibrosis, even when patients with renal failure present complications in other organs such as heart. In previous studies in renal injury models, long-term treatment with Ang-(1–7) decreased the production of pro-inflammatory cytokines, macrophage infiltrate, caspases cleavage and blood pressure, as well as attenuating Ang II levels meliorating oxidative stress and renal fibrosis (Lu et al., 2017; Zaman and Banday, 2022). Moreover, treatment was able to increase the activity of enzymes involved in collagen degradation, and improved heart function accompanied by a reduction in cardiac fibrosis (Li et al., 2009). Interestingly, in renal failure models, Mas receptor deficiency inhibited NF-κB phosphorylation leading to reduction in MCP-1 and IL-6 cytokines and thus attenuating the fibrotic condition. Similar results were observed when wildtype experimental models were infused with short-term Ang-(1–7) treatment, suggesting that in some conditions, lack of signaling by Mas could prevent renal inflammation (Esteban et al., 2009).

One of the most studied actions of Ang-(1–7) is in cardiac remodeling. Many authors have confirmed that Ang-(1–7) has some effect in at least one of the components of cardiac remodeling. Grobe *et al.* demonstrated reduced myocyte hypertrophy, interstitial fibrosis and TGF-β levels in response to Ang-(1–7) in an Ang II-induced rat model of hypertension and cardiac remodeling (Grobe et al., 2007). Studies with transgenic mice overexpressing Ang-(1–7) in the heart have shown that in the presence of Ang II, hypertrophy and fibrosis of the left ventricle is reduced, as well as expression of TGF-β1 (Mercure et al., 2008). In cardiac fibrosis, exposure of Ang II-stimulated cardiac fibroblasts to Ang-(1–7) inhibited collagen synthesis and expression of endothelin-1 and leukaemia inhibitory factor (Iwata et al., 2005). It has also been described that Ang-(1–7) normalizes the decreased levels of MMP in Ang II-stimulated cardiac fibroblasts and myocytes (Pan et al., 2008). In addition, Grobe *et al.* (2006) showed that Ang1-(1–7) was able to prevent interstitial fibrosis by decreasing collagen deposition in the deoxycorticosterone acetate (DOCA) salt hypertensive rat model (Grobe et al., 2006). In nephrectomised mice Ang-(1–7) prevented left ventricular remodeling and diminished interstitial fibrosis by reducing the levels of TGF-β and increasing MMP2 and 9 (Li et al., 2009). Ang-(1–7) also decreased the expression levels of inflammatory cytokines and suppressed oxidative damage (Li et al., 2009) (Figure 3). It has been demonstrated that oxidative stress, vasoactive peptides, and inflammation can induce DNA damage, mitochondrial dysfunction, and protein misfolding leading to senescence and cell death (Erusalimsky, 2009). Endothelial senescence has been associated with cardiovascular diseases and atherosclerotic lesions (Mogi, 2020). Regarding Ang-(1–7), Romero et al. (2019), studied the heptapeptide role in endothelial cells senescence-induced by RAS or non-RAS mechanism. They found that Ang-(1–7) antagonized the cell senescence triggered by Ang II and IL-1β, which were blunted by A779 (Mas antagonist). The pro-inflammatory phenotype also was reduced after peptide treatment, which included a reduction in adhesines such as ICAM-1, VCAM, and IL-6 secretion. Additionally, the nuclear factor-erythroid 2-related factor 2 (Nrf2) and heme oxygenase-1 (HO-1) levels were augmented after Ang-(1–7) and inhibited when these pathways were blocked. These data suggest that Ang-(1–7) could prevent Ang II and IL-1β senescencent-induced effects by reducing oxidative stress by MasR/Nrf2/HO-1 axis (Romero et al., 2019). As mentioned above, oxidative stress and cytokine production can trigger cell senescence. Several reports on ROS dysregulation are related to the development of fibrosis diseases (Li et al., 2020). Due to that in fibrosis, there is low-grade chronic inflammation, which triggers excessive ROS production and may promote TGF-β synthesis leading to fibroblast activation with consequent ECM deposit accumulation. Hence, it could be interesting to study the pathways related to aging and fibrosis development.

### 3.2 Angiotensin-(3–8)

Ang-(3–8), also known as Ang IV, is a hexapeptide generated through the cleavage of Ang III by aminopeptidase N. This peptide of only six amino acids long has been shown to have vasodilatory and inflammatory actions in different organs. The role of Ang-(3–8) in inflammation was described for the first time in 2005. The capacity of this peptide to activate the transcription factor NF-κB and positively regulate inflammation was demonstrated in vascular smooth muscle cells through the AT_4_R receptor (Esteban et al., 2005). On the contrary, studies have emerged establishing Ang-(3–8) having anti-inflammatory effects. In human endothelial cells, the capacity of Ang IV to mediate the expression of Macrophage Migration Inhibitor, a proinflammatory cytokine associated with the production of TNF-α, IL-1β and IL-6, was observed (Zhong et al., 2008). In murine macrophages, Nikolaou *et al.* (2014), evaluated the effects of Ang IV on the NF-κB pathway, where a lack of expression of proinflammatory genes such as ICAM-1 and TNF-α was observed, which could indicate differential effects of this peptide depending on cell lineage (Nikolaou et al., 2014). In a murine model of abdominal aortic aneurysm, treatment with Ang IV markedly reduced the infiltration of macrophages and proinflammatory cytokines (Kong et al., 2015). Furthermore, in cardiac ischemia-reperfusion (I/R) injury, Ang-(3–8) infusion managed to suppress the expression of VCAM-1, TNFα, MMP-9, and NF-κB proteins (Figure 3) (Park et al., 2016). Similar results were described in rats with cerebral hypoperfusion, where in addition to observing anti-inflammatory effects, it was discovered for the first time that this effect was induced in a dose-dependent manner (Wang et al., 2018). More recently, in mice, it was demonstrated how Ang IV was able to protect against acute myocardial infarction by inhibiting inflammation (Bai et al., 2021).

In a fibrotic context, little is still known in relation to Ang IV. However, some studies have demonstrated an interaction between this peptide and fibrotic pathologies. In a study performed in kidney cells, the capacity of Ang IV to induce the expression of plasminogen activator inhibitor-1 mRNA, a protein involved in fibrosis inhibition and progression of fibrotic events, was demonstrated (Gesualdo et al., 1999). Ang-(3–8) also induced interstitial fibrosis and cardiac deterioration in adult mouse hearts when bound to the AT_1_R receptor (Ainscough et al., 2009). Conversely, Ang IV dose-dependently downregulated Fox01-mediated fibrosis when bound to the AT4 receptor in a mouse model of diabetic cardiomyopathy (Zhang M. et al., 2021) (Figure 3).

### 3.3 Angiotensin-(1–9)

Ang-(1–9) is a nine amino acid peptide that results from the metabolism of Ang I by ACE2 cleavage of the terminal amino acid (Donoghue et al., 2000). Although ACE2 is the main enzyme to form Ang-(1–9), carboxypeptidase A and cathepsin A can also produce this peptide (Jackman et al., 2002; Garabelli et al., 2008). These reports also show that formation of Ang-(1–9) from Ang I is the main pathway in cardiomyocytes (Kokkonen, et al., 1997; Garabelli et al., 2008). As already mentioned, once Ang-(1–9) is formed ACE cleaves the two last amino acids (phenylalanine and histidine) generating the active peptide Ang-(1–7). Ang I is the precursor to Ang-(1–9) through ACE2 (Figure 1). Although little is known about it, Ang-(1–9) has gained relevance as a counterregulatory active peptide. Ang-(1–9) binds mainly to the AT_2_R (Flores-Muñoz et al., 2011), exerting biological effects in organs such as kidney, lung and mainly heart.

Few studies have described Ang-(1–9) in an inflammatory context (Figure 2). In rats with diabetic heart disease, subcutaneous administration of this peptide normalized the levels of several proinflammatory cytokines, including TNF-α and IL-1β through the AT_2_R (Zheng et al., 2015). In DOCA-salt hypertensive rats, infusion of Ang-(1–9) decrease monocyte infiltration in heart, aorta and kidney tissue, reducing inflammation in these rats (Gonzalez et al., 2018). Similar results were also observed in a rat model of pulmonary hypertension, where treatment with Ang-(1–9) reduced plasma TNF-α, MCP-1, IL-1β, and IL-6 (Cha, et al., 2018). Furthermore, in prostate cancer cells Ang-(1–9) negatively regulated the expression of NF-κB1 and NF-κB2, modulating these inflammatory pathways (Domińska et al., 2020)

Within the fibrotic context Ang-(1–9) was shown to be capable of reducing cardiac fibrosis through the AT_2_R, by reducing the presence of type I collagen in spontaneously hypertensive rats (Flores-Munoz et al., 2012). Similarly in rats with Ang II-induced hypertension, this peptide was able to reduce the presence of collagen type I, in addition to reducing the expression of TGF-β (Ocaranza et al., 2014). These results were also observed in diabetic rats where collagen I and TGF-β mRNA expression was also decreased (Zheng et al., 2015). Adenoassociated delivery of Ang-(1–9) in mice infarcted hearts, reduced septal and perivascular fibrosis, as well as expression of MMP-12, described as promoter of fibrosis (Fattah et al., 2016). Furthermore, Fattah *et al.* (2016), described that Ang-(1–9) delivery, while reducing fibrosis, it also reduced acute rupture by stabilizing and thickening myocardial infarction scar, suggesting Ang-(1–9) remodeling modulation during scar evolution. Gonzalez *et al.* (2018), described Ang-(1–9) as having a protective role in hypertensive end-organ damage, by demonstrating reduction of collagen deposition and myofibroblast in heart, kidney and aorta when infused in DOCA-salt hypertensive rats (Gonzalez et al., 2018). Additionally, in rats with pulmonary hypertension the antifibrotic effects of Ang-(1–9) were replicated. Treatment with Ang-(1–9) in an monocrotaline induced pulmonary hypertensive model reduced pulmonary damage via the AT_2_R (Cha et al., 2018) (Figure 3).

### 3.4 Alamandine

Alamandine is a recently discovered heptapeptide derived from Angiotensin A, very similar to Ang-(1–7), differing only by presenting alanine instead of aspartate in the N-terminal domain (Lautner et al., 2013). Due to this similarity, alamandine has been shown to have similar actions to Ang-(1–7). However, these actions have been associated with its interaction with a different receptor, the Mas-related G protein-coupled receptor, member D (MrgD) (Lautner et al., 2013) (Figure 2). Within the inflammatory context, *in vitro* experiment showed that alamandine was able to dose-dependently regulate the degranulation of MMP-9 and myeloperoxidase in mouse neutrophils, suggesting an anti-inflammatory role (Da Silva et al., 2017). Later, in a murine model of cardiac dysfunction associated with sepsis, this peptide was able to prevent myocardial inflammation, by preventing the activation of ERK, JNK and P38 (Li et al., 2018). Furthermore, in mice subjected to a transverse aortic constriction procedure, it was observed that alamandine was able to decrease the expression of proinflammatory genes MCP-1, TNF-α, IL-1β and contribute to resolution with an increased expression of MRC1 and FIZZ1 (Morais Silva, 2020). Similar results were observed in a myocardial ischemia-reperfusion injury model where alamandine reduced the levels of TNF-α, IL-1β, IL-6 and NO, and protected cardiomyocytes by inhibiting the activation of NF-κB (Song et al., 2019). Therapeutic administration of this peptide decreased the number of neutrophils and M1 macrophages in a model of LPS-induced inflammation (De Carvalho Santuchi et al., 2019). Recently, alamandine was able to reduce doxorubicin-induced cardiotoxicity in rats, by counteracting the elevation of proinflammatory cytokines (Hekmat et al., 2021). In the kidney, this peptide also demonstrated anti-inflammatory actions by alleviating kidney injury by inhibiting PI3K/AK and MAPK pathways (Hu et al., 2021). Furthermore, alamandine was shown to have a protective role in a stroke model by reducing the expression of proinflammatory cytokines (TNFα, IL-1β, IL-6) (Figure 2; 3) (Gonçalves et al., 2022). Such protective effects were also observed in a collagen induced arthritis model (Ding et al., 2022).

Alamandine has also been described as having antifibrotic effects in different pathologies. Oral treatment of alamandine to spontaneously hypertensive rats resulted in decrease of collagen I, III and fibronectin expression of (Lautner et al., 2013; Liu et al., 2017). Similarly, administration of alamandine regulated vascular remodeling of ascending aorta by diminishing aortic fibrosis, collagen deposition, MMP activity and TGF-β expression in a murine model of transverse aortic contrition, as well as several important pro inflammatory genes (MCP-1, TNF-α, IL1-β) (De Souza-Neto et al., 2019). These effects are mediated through the MrgD receptor (Yang et al., 2020). In a subsequent study, when analyzing cardiac remodeling, alamandine administration reduced collagen deposition, MMP-2 and TGF-β expression in the left ventricle, induced by aortic constriction. Additionally, alamandine prevented ERK1/2 and AMPKα phosphorylation, suggesting important effects of alamandine in cardiac remodeling regulation (Morais Silva, 2020). Furthermore, Wang *et al.* (2023), suggested that alamandine antifibrotic effects on TGF-β activated fibroblast could be mediated by decreasing glycolysis through Parkin/CSF mitophagy (Wang et al., 2023). NF-κB and JNK pathways have been linked to alamandine actions. In a rat model of cardiac ischemia-reperfusion injury, the administration of this peptide reduced the presence of fibrotic markers, increasing the phosphorylation levels of ERK and JNK, while NF-κB decreased (Song et al., 2019). Another mechanism associated with the effects of alamandine was observed in liver and lung, where the peptide attenuated the presence of fibrosis by attenuating autophagy activated by ROS-dependent reactive oxygen species (Huang et al., 2020; Gong et al., 2022; Zhao et al., 2022). (Figure 3).

### 3.5 Other functional peptides of the counter-regulatory axis

Currently, as part of the counter-regulatory RAS there are several other angiotensin peptides, but their role in inflammation or fibrosis has not been yet described. Within these, Ang III is a heptapeptide produced by Ang II cleaved in the Asp N-terminal residue, mediated by aminopeptidase A, and has been demonstrated to have high affinity to AT_1_R but shows selectivity to AT_2_R (Bosnyak et al., 2011). Ang III can induce NF-κB phosphorylation in mesangial cells and increase MAPK phosphorylation on VSMC, inducing proliferation. These effects are mainly mediated through the AT_2_R (Lorenzo et al., 2002; Alanazi and Clark, 2020). In addition, low concentrations of Ang III perfusion have been demonstrated to stimulate stretch-induced atrial natriuretic peptide (ANP) secretion. ANP secretion was abolished when the AT_2_R was blocked but not AT_1_R or Mas. Interestingly, such an effect was also blocked by pretreatment with PI3K, Akt or PKG inhibitor, showing that ANP secretion may be stimulated by PI3K–Akt–PKG signaling pathway (Park et al., 2013). Moreover, intrarenal treatment with Ang III augmented urine sodium excretion in rats and increased AT_2_R expression, effect that was abolished by an AT_2_R antagonist (Padia et al., 2009). In addition, Ang III treatment produced a significant reduction of diastolic blood pressure in spontaneously hypertensive rats (Matsufuji et al., 1995). However, in another study, Ang III infusion failed to increase urine sodium excretion rate and AT_2_R upregulation suggesting that the natriuretic effect is mediated by AT_2_R signaling (Padia et al., 2009; Kemp et al., 2019). These data together suggest that Ang III could have an important pathologic role in renal diseases; however, further studies are needed to understand its clinical implications.

On the other hand, Ang-(3–7) is formed by cleavage of the Pro-Phe amino acid residue from Ang IV by carboxypeptidase P (Wright et al., 2012). Early studies that evaluated Ang-(3–7) showed its pressor effect in rats (Ferreira A. J. et al., 2007). Subsequently, the role has been studied *in vitro* in prostate epithelial cells, observing that Ang-(3–7) incubation led to upregulation of MIK67, NF-κB gene expression, and mobility was increased (Domińska et al., 2020). Additionally, Ang-(3–7) administration reduced heart weight and cardiac hypertrophy gene markers ANP, BNP, and β-MHC. Furthermore, the peptide treatment inhibited collagen I, collagen III, and fibronectin expression in hearts’ mice and cardiac fibroblast cells. These results indicated that Ang-(3–7) could attenuate cardiac hypertrophy and prevent fibrosis development in experimental cardiac remodeling model (Zhang Y. et al., 2021).

Ang-(3–4) is one of the shorter peptides in the counter-regulatory RAS that has been shown to have some effects. It is produced from Ang III metabolism in plasma (Matsufuji et al., 1995) or by Ang-(1–7) cleavage in the kidney (Axelband et al., 2009). The first studies showing some effect of this peptide demonstrated a role as an antihypertensive, antiproliferative, and vasodilatory agent (Matsufuji et al., 1995; Matsui et al., 2005; Dias et al., 2017). In hypertensive models, it has been found that oral administration of Ang-(3–4) produced a reduction in systolic blood pressure, as well as urinary sodium excretion increased and AT_1_R downregulated in the renal proximal tubule of young rats (Saito et al., 1994; Luzes and Crisóstomo, 2021). Interestingly, the dipeptide depressor effect was not significant in aged mice (Matsui et al., 2003). In addition, *in vitro* studies show that Ang-(3–4) can also inhibit Ang II induced-human VSMC proliferation through intracellular Ca^2+^ suppression (Matsui et al., 2005). In a subsequent study, it was demonstrated ACE inhibition by dipeptide’s stimulus led to a reduction of Ang I-produced contraction of the aortic rings in rats (Vercruysse et al., 2008). Additionally, Ang-(3–4) effects are also investigated in overweight and undernutrition. In an overweight rat model, it was found that Ang-(3–4) treatment reduced blood pressure and hepatorenal index, suggesting that the peptide could prevent lesion formation induced by lipid deposits (Crisóstomo et al., 2022). Moreover, it has been found that in aging overweight and undernutrition juvenile rats, ACE2 levels decrease. Interestingly, after Ang-(3–4) administration, ACE2 levels are increased in overweight rats but not in rats with undernutrition conditions (Luzes and Muzi-Filho, 2021). In contrast, in a recent study, Pereira-Acácio et al. showed that a multi-deficient diet led to high blood pressure, and it could be associated with the development of hypertension (Pereira-Acácio et al., 2022). In this sense, they demonstrated that Ang-(3–4) treatment is capable of normalizing systolic blood pressure (Pereira-Acácio et al., 2022).

Finally, another recently studied peptide is Ang-(1–5). This pentapeptide is derived from Ang-(1–7) *in vitro* metabolism by the ACE enzyme, which hydrolyzes its Ile- His amino acid residues (Chappell et al., 1998). The most researched effects of pentapeptide are focused on the cardiovascular system. Roks *et al.* (1999), demonstrated that *in vitro* stimuli with Ang-(1–5) inhibited ACE activity from human plasma but is not capable of affecting arterial contractions (Roks et al., 1999). In addition, in isolated beating atria Ang-(1–5) stimulation induced augmented ANP secretion that was attenuated after inhibition of Mas, PI3K, Akt, and NOS, suggesting these pathways could be involved in ANP secretion stimulated by pentapeptide (Yu et al., 2016). Subsequently, the effect of Ang-(1–5) in Sprague-Dawley rats with I/R injury was evaluated. In this study, rats’ hearts were infused with low doses of Ang-(1–5) for 10 min prior to ischemic induction, further, they used AT_1_R, AT_2_R, and Mas antagonists. The Ang-(1–5) infusion ameliorated I/R-induced changes in left ventricular end-diastolic and developed pressure, cardiac infarct size and ANP secretion. These effects were abolished by the treatment with Mas antagonist, but not by AT_1_R or AT_2_R blockers. Moreover, the pentapeptide treatment also reduced pro-apoptotic proteins such as Bax, Caspase-3, and 9. However, anti-apoptotic proteins such as Bcl-2, and antioxidant enzymes such as Mn-superoxide dismutase, catalase, and HO-1 were increased. These effects were blocked when Mas was inhibited (Park, et al., 2021). Myocardial ischemic/hypoxia models are characterized by a gradual decrease in ANP secretion and changes in the profile of antioxidant enzymes and ROS production that lead to an increase in cell death (Park et al., 2021). Hence, this data together suggests that Ang-(1–5) treatment could attenuate myocardial damage in this pathology. Despite the recent evidence about the effects of these peptides, most reports are focused on their anti-hypertensive role. However, taken together all the available data, it could suggest that the upregulation of antioxidant pathways will lead to a reduction in ROS production with a consequent decrease in tissue damage to avoid chronic inflammation and fibrosis development.

## 4 AT2R and MAS agonists

### 4.1 Compound 21

Compound 21 (C21) is the first non-peptide agonist of the AT_2_R. This agonist was synthesized in 2004 by Yiqian Wan, and through a binding assay they demonstrated the selective affinity of C21 for AT_2_R. Additionally and most importantly, this new C21 had vasopressor effects in spontaneously hypertensive rats (Wan et al., 2004). The synthesis of this compound has facilitated the study of the specific effects of AT_2_R. Antihypertensive, proangiogenic, antifibrotic and anti-inflammatory effects in different organs have been attributed to the interaction of C21 and AT_2_R.

The anti-inflammatory actions of C21 with the AT_2_R interaction have been studied in different pathologies. In a model of acute myocardial infarction in Wistar rats, the intraperitoneal administration of C21 was able to stimulate AT_2_R, observing improvement in cardiac function and reduction of the scar in the post-infarction heart (Kaschina et al., 2008). In addition to these results, a null expression of p38 MAPK and p44/42 MAPK was observed. These MAPKs are traditionally involved in processes such as proliferative, apoptotic, and inflammatory. However, there is also evidence of opposite effects to those mentioned above. This association was demonstrated with evidence of anti-inflammatory actions, where C21 significantly suppressed the expression of cytokines (MCP-1, IL-1, IL-2, IL-6), so C21 contributed to the preservation of normal physiology after of myocardial infarction (Kaschina et al., 2008). Sampson et al., studied the involvement of C21 in TNF-α-induced inflammation in HUVEC, monocyte activation and in aortas of C57BL/6 mice. *In vitro* experiments indicated that C21 reduced TNF-α-induced expression of ICAM-1, CCL2, and IL-6 genes and proteins. In addition to reducing increased monocyte adhesion by 40% and reduced NFkB-p65 translocation from cytoplasm to the nucleus. These results were extrapolated to *in vivo* experiments, where treatment with C21 showed similar results (Sampson et al., 2016).

In renal pathological conditions, C21 counteracts most of the effects of the AT_1_R. Matavelli *et al.* (2011), evaluated early renal inflammation in renovascular hypertension. In Sprague-Dawley rats treated with C21 for 4 days, a reversal of the early inflammatory stage was observed, with reduction of the proinflammatory markers TNF-α, IL-6 and TGF-β1 in kidney tissue (Matavelli, et al., 2011). Another study in 2015 evaluated the involvement of C21 in a model of early diabetes in rats, where one of its main objectives was to study the effects of this compound on kidney inflammation. In this case, in addition to seeing a marked reduction in IL-6, C21 also reduced TNF-α and markers of oxidative stress (Matavelli et al., 2015). Years later, Jabber *et al.* (2023), demonstrated in a murine model of sepsis-induced renal inflammation that the modulation of C21-induced inflammation was mediated by the modulation of the PI3K/AKT signaling pathways (Jabber, et al., 2023).

In the lung, C21, as in the heart and kidney, proved to have anti-inflammatory potential. In experiments carried out in mice with pulmonary inflammation induced by bleomycin, the contribution of C21 as a pulmonary anti-inflammatory was confirmed for the first time, observing a reduction in cellular infiltrate in bronchoalveolar fluid (Du et al., 2009). In subsequent studies, carried out by Menk *et al.* (2018), it was demonstrated that, in this organ, C21 could reduce not only the cellular infiltrate, but also the expression of TNF-α and IL-6 (Menk et al., 2018). Similar results were observed in Chronic obstructive pulmonary disease (COPD) induced by cigarette smoke, showing a reduction in proinflammatory cytokines and, also, the inhibition of components of the NF-κB signaling pathway in alveolar macrophages (Mei et al., 2022). Currently, with the emergence of the COVID-19 pandemic, the study of acute respiratory distress syndrome (ARDS) has gained relevance. In this sense, Chen *et al.* (2024), studied the relationship of C21 in ARDS, where favorably, this compound was able to resolve the inflammatory stage, with a marked reduction of CCL-2, IL-6 through NF-κB (Chen et al., 2024).

C21 has been shown to have antifibrotic actions in various cardiac pathologies. In 2012, Rehman *et al.* (2012), studied the involvement of C21 in the development of hypertension and vascular damage in stroke-prone spontaneously hypertensive rats. In these rats administered with C21 for 6 weeks, a decrease in fibrotic and hypertensive events was observed, associated with the reduction of the stiffness of the mesenteric artery, the decrease in myocardial interstitial collagen type I/III and aortic oxidative stress, infiltrate of inflammatory cells and fibronectin (Rehman et al., 2012). Subsequently, in rats with myocardial infarction, in addition to observing the ability of C21 to improve arterial stiffness and reduce collagen, a decrease in the extracellular matrix metalloproteases MMP2 and MMP9 and transforming growth factor B was observed (Lauer et al., 2013). Similarly, in another study of cardiac hypertrophy induced by high salt intake, C21 was able to replicate previous results (Dopona et al., 2019). In rats with Ang II-dependent hypertension, a sustained reduction in myocardial perivascular fibrosis was observed after 1 week of intraperitoneal administration of C21 (Castoldi et al., 2016).

In *in vivo* and *in vitro* experiments carried out in ydiabetic rats and mesangial cells, Koulis *et al.* (2015), demonstrated that the activation of AT_2_R by C21 reduced the gene and protein expression of TGF-β1, CTGF, smooth muscle alpha actin and MMP-2 at the renal level. Furthermore, the effect of C21 on the extracellular matrix induced a reduction in type I and IV collagen (predominant collagen isotypes in renal fibrosis) (Koulis et al., 2015). In rats with cyclosporine-induced nephropathy, C21 reduced these same fibrotic markers, in addition to reducing glomerular and tubulo-interstitial fibrosis and macrophage infiltrate (Castoldi et al., 2016).

The presence of fibrosis in the lung can lead to decreased lung capacity, respiratory failure, and other complications such as heart problems. C21 has also been studied to treat fibrotic lung pathologies, with promising results. In a model of pulmonaryb hypertension in rats, induced by monocrotalin, the intraperitoneal administration of C21 decreased the presence of interstitial and perivascular collagen I, in addition to reducing the gene expression of profibrotic cytokines (TNF-α and IL-1β) (Bruce et al., 2015). Similarly, in a bleomycin-induced lung injury model in rats, administration of C21 at the same concentration reduced lung collagen accumulation, attenuated gene expression of fibrotic markers (Col 1, Col 3, Ctgf, Mmp12, Timp1, IL-13) (Rathinasabapathy et al., 2018). Recently, in a model of pulmonary hypertension induced by hypoxia in rats, C21 was administered intraperitoneally at two different doses. It was shown that this compound was able to reduce the presence of collagen fibers significantly at the dose of 20 mg/kg but not with the dose of 2 mg/kg (Tornling et al., 2023).

### 4.2 AVE 0991

The compound AVE 0991 (AVE) was first described in 2002 by Wiemer *et al.* (2002). In this study they demonstrated that AVE 0991 mimicked the effects of Ang-(1–7) on endothelium, reducing the) release of nitric oxide, maintaining endothelial function, and reducing vascular injury (Wiemer et al., 2002), through the G protein-coupled receptor MAS (Pinheiro et al., 2004).

In heart diseases, AVE has been shown to induce beneficial effects for the resolution of pathologies. Zhou *et al.* (2019), in a murine model of transverse aortic constriction, tested the effects of AVE in combination with intraperitoneal captopril. Coadministration of these compounds completely prevented macrophage infiltration in the aortic adventitia. However, there was an accumulation of cells still present (Zhou et al., 2019). In another study of Ang II-induced hypertension in rats, AVE treatment in combination with alamandine reduced inflammatory stress related to the increase in MCP-1 (Tanrıverdi et al., 2023).

In an atherosclerosis model in knockout mice, AVE administered as treatment for 4 months, reduced the infiltration of proinflammatory cells MCP-1, IL-6, IL-12 and SAA (Jawien et al., 2012). Skiba et al. (2017), demonstrator that in this model, AVE 0991, inhibited inflammation in perivascular adipose tissue by reducing the expression of cytokines IL-1β, TNF-α, MCP-1 and CXCL10, and the differentiation of M2 macrophages to the M1 phenotype (Skiba et al., 2017).

This non-peptide agonist has also an anti-inflammatory effect in pulmonary pathologies. Rodrigues-Machado *et al.* (2013), evaluated the effects of AVE in a model of chronic asthma induced with ovalbumin in BALB/c mice. After administration of AVE for 28 days, this treatment was able to prevent the development of pulmonary and airway vascular remodeling, in addition to reducing the inflammatory response with a marked reduction in cytokine release (IL-5) in bronchoalveolar fluid and lung homogenates (Rodrigues-Machado et al., 2013). Subsequently, in a similar model it was observed that the anti-inflammatory effect (reduction of macrophages, MCP-1, MAPK) occurred through the MAS receptor expressed in the bronchial epithelium. Furthermore, these actions were attributed to the inhibition of the JNK signaling pathway (Hong et al., 2021).

In a fibrotic context, although little is known to date, AVE has had favorable implications in the resolution of different pathologies that develop this alteration. In a model of cardiac dysfunction in rats, induced by isoproterenol, the intraperitoneal administration of AVE attenuated the deposition of collagen in the left ventricle, in addition to reducing the presence of collagen type I, collagen III and fibronectin in the heart (Ferreira P. M. et al., 2007). In rats with myocardial infarction, AVE also attenuated the expression of collagen I and III, in addition to inhibiting the expression of TGF-β and TNF-α (Zeng et al., 2012). In a model of renal hypertension in rats, intragastric administration of AVE for 28 days was shown to be capable of reducing collagen deposition in renal tissue (Cunha et al., 2013; Cao et al., 2019). Cao *et al.* (2019), in a model of pulmonary fibrosis caused by LPS-induced ARDS, evaluated the effects of AVE, where they demonstrated that the application of this compound as an intraperitoneal treatment for 20 days was capable of attenuating lung injury by reducing the presence of collagen I fibers, the levels of TGF-β in bronchoalveolar fluid and plasma, the protein expression of E-cadherin and vimectin and the phosphorylation of Src kinase (Cao et al., 2019).

## 5 Translational relevance

Peptides of the counterregulatory axis of the renin-angiotensin system are likely to play an important role in the control of inflammatory and fibrotic diseases, as supported by many *in vitro* and *in vivo* studies. Still, to date, existing studies remain limited in the clinical context.

As we have seen in this review, Ang-(1–7) is one of the most studied peptides, so there are several reports that evaluate its actions as clinical treatment. Although several of these studies are not directly in inflammatory or fibrotic diseases, it is relevant to mention them.

Ueda *et al.* (2000), evaluated the vascular effects of Ang-(1–7) in human forearm resistant vessels, where intra-arterial infusion of this peptide reduced the increase in blood flow caused by Ang II in normotensive patients (Ueda et al., 2000). Furthermore, in normotensive subjects, Ang-(1–7) was able to enhance the vasodilatory effects of bradykinin, reducing forearm blood flow by 10 percent (Ueda et al., 2001). Later, these same actions were evaluated in normotensive and hypertensive subjects, observing an increase in vasodilation in both conditions (Sasaki et al., 2001). More recently, in healthy normotensive postpartum women with preeclampsia, Ang-(1–7) enhanced the endothelium-dependent vasodilatory response, attenuating Ang II-mediated constriction (Stanhewicz and Alexander, 2020). The main beneficial effects described on these peptides are as vasodilators and anti-hypertensives. Hence, the improvement in blood flow could have anti-inflammatory effects by reducing the accumulation of inflammatory cells and increasing the arrival of anti-inflammatory factors, which could promote the resolution of this response. On the other hand, the reduction of inflammation could regulate the elevation of pro-fibrotic factors, which stimulate the excessive synthesis of extracellular matrix components and the proliferation of fibroblasts, reducing the progression of fibrosis.

To our knowledge, there are very few clinical trials that have evaluated the implications of Ang-(1–7) in an inflammatory context. In confirmed COVID-19 patients, the effects of plasma with Ang-(1–7) were evaluated, observing an improvement in the clinical status of the patient (Onal et al., 2022). Importantly, two independent pilot clinical trials aimed to determine the safety of human use of TXA-127 [a pharmaceutically formulated Ang-(1–7)] (Wagener et al., 2022), or Ang-(1–7) (Valle Martins et al., 2022) delivered these peptides intravenously to COVID-19 patients, showing a safe use of both molecules, and suggesting the potential clinical use as treatment for severe COVID-19.

Other peptides of the counterregulatory axis have not been evaluated in clinical studies, however the knowledge generated so far in cells and animals could lay the foundations for future research in humans focused on the development of anti-inflammatory and antifibrotic treatments.

## 6 Conclusion

In conclusion, the counterregulatory axis of the renin-angiotensin system has become an important object of study for more than 2 decades. The components of this axis offer a variety of effects such as antihypertensive, anti-inflammatory and antifibrotic. In the field of research, the evaluation of these components has progressed from structural observation through histological techniques to the evaluation of gene and protein expression. Several studies have described some mechanisms of action by which the components of the counterregulatory axis act in the regulation of inflammation and fibrosis. These mechanisms have focused on the evaluation of signaling pathways such as NF-κB, JAK/STAT and JNK.

Therefore, the peptides of the counterregulatory axis of the renin-angiotensin system, as well as their non-peptide analogues, offer new options for the therapy of pathologies that have inflammatory and fibrotic stages. However, several challenges remain, including:

1. There is literature that describe controversial evidence on some of the peptides regarding their anti-inflammatory effects.
2. The understanding of the mechanisms of action is still very limited, with little literature that supports the signaling pathways involved in its effects.
3. Most of the effects described so far are evaluated in *in vivo* or *in vitro* models and in numerous cases still require clinical research to demonstrate the reproducibility of their results in humans.

In future research, it is expected that by improving the understanding of the action of this axis and its relationship with the progression of diseases, these peptides can be applied clinically as treatments that help reduce the alarming response of diseases to less serious stages.
